# IL-6/GP130/JAK/STAT3 Pathway Activation in Pancreatic Ductal Adenocarcinoma and Its Association With Clinical Features: Protocol for a Retrospective Cross-Sectional Study

**DOI:** 10.2196/76811

**Published:** 2025-12-15

**Authors:** Claudia Mellenthin, Fabien Schaller, Lucie Vignot, Sarah Peisl, Rita Vesce, Irene Esposito, Carmen Gonelle-Gispert, Cornelia Schneider, Michel Adamina, Leo Hans Bühler

**Affiliations:** 1 Department of Surgery, Hospital Fribourg, Fribourg, Switzerland; 2 Department of Medical and Surgical Specialties, Faculty of Science and Medicine, University of Fribourg, Avenue de l'Europe 20Fribourg, 1700, Switzerland, 41 264963333; 3 Department of Oncology, Hospital Fribourg, Fribourg, Switzerland; 4 Department of Visceral Surgery and Medicine, Inselspital, Bern University Hospital, University of Bern, Bern, Switzerland; 5 Institute of Pathology, Düsseldorf University Hospital, Düsseldorf, Germany; 6 Clinical Pharmacy and Epidemiology, Department of Pharmaceutical Sciences, University of Basel, Basel, Basel-City, Switzerland; 7 Basel Pharmacoepidemiology Unit, University Hospital Basel, Basel, Basel-City, Switzerland

**Keywords:** pancreatic cancer, IL-6, STAT3, survival, inflammation, interleukin 6, signal transducer and activator of transcription 3

## Abstract

**Background:**

The proinflammatory cytokine interleukin 6 (IL-6) contributes to pancreatic tumorigenesis by activating the Janus kinase (JAK)–signal transducer and activator of transcription 3 (STAT3) signaling cascade. Targeting the IL-6/glycoprotein 130 (GP130)/JAK/STAT3 axis may therefore represent a promising therapeutic approach for pancreatic adenocarcinoma.

**Objective:**

This study investigates the activation of the IL-6/GP130/JAK/STAT3 pathway in archived biopsy samples from patients with pancreatic adenocarcinoma, quantifying its expression in both tumor and stromal compartments. In addition, potential associations between pathway activation and clinical outcomes are explored, including tumor stage, patient survival, and metabolic parameters such as diabetes, use of oral antidiabetic drugs, insulin therapy, and hemoglobin A_1c _levels.

**Methods:**

We will conduct a retrospective cross-sectional analysis of patients with pancreatic adenocarcinoma treated at the Cantonal Hospital of Fribourg, Switzerland, between 2010 and 2025. Eligible cases are identified through tumor board records. Archived pathology specimens will be immunostained using a phospho-STAT3 antibody to assess GP130 receptor activation. The percentage of positively stained cells will be quantified separately for tumor tissue, stroma, and adjacent normal tissue using QPath software. Sample size calculations are based on the assumption that patients with higher IL-6 pathway activation have poorer prognoses. Detecting a 5% difference in activation requires 84 patients per group for 90% power or 104 per group for 95% power. Diabetes will be analyzed as a dichotomous variable, while IL-6/GP130/JAK/STAT3 activation will be treated as a continuous variable. Descriptive statistics will summarize pathway activation and clinical variables. Mean differences between groups will be compared, and survival outcomes will be evaluated using Cox proportional hazards models. Hazard ratios will be calculated for all metabolic parameters to assess potential associations between pathway activation and diabetes. All analyses will include 95% CIs, with missing data reported transparently.

**Results:**

This project is supported by a research grant from the Cantonal Hospital of Fribourg (HFR 2/2021 and 4/2022). It was approved by the Ethics Committee of Bern, Switzerland (project 2024‐01215). As of October 12, 2025, clinical records from 150 patients have been identified. Screening of additional patient records from 2024 to 2025 is ongoing.

**Conclusions:**

This study represents one of the first translational investigations of IL-6/GP130/JAK/STAT3 signaling in pancreatic adenocarcinoma. By correlating molecular pathway activation with clinical and metabolic features, it will provide new insights into disease heterogeneity and support the development of personalized, pathway-targeted therapeutic strategies.

## Introduction

### Clinical Perspective

Pancreatic ductal adenocarcinoma (PDAC) remains one of the solid cancers with the worst prognosis. It has become the third leading cause of cancer-related death, largely because of late discovery and unresectability but also because of the lack of highly efficient chemo-, immuno-, and radiotherapies [1]. The ESPAC (European Study Group for Pancreatic Cancer) trials [2-5] showed that chemotherapy improves survival in the adjuvant and neoadjuvant setting in borderline resectable patients. Still, survival remains worse than in most other cancers. While immunotherapy has been a game changer in many cancers, there has not been such a breakthrough for pancreatic cancer so far [6].

An overall survival of 10% at 5 years has not been exceeded in most countries [7]. In Switzerland, overall survival has recently increased to 13% for the period, whereas incidence also increased by more than 10% compared with the period of 2008‐2012 [8].

Given that chemotherapy administration is frequently constrained by rapid patient deterioration, including weight loss and impaired glucose metabolism, we aim to investigate the association between these clinical features and the tumor stage/prognosis with the inflammatory interleukin 6 (IL-6)/glycoprotein 130 (GP130)/Janus kinase (JAK)/signal transducer and activator of transcription 3 (STAT3) signaling pathway.

### Pathophysiology

**Figure 1.**
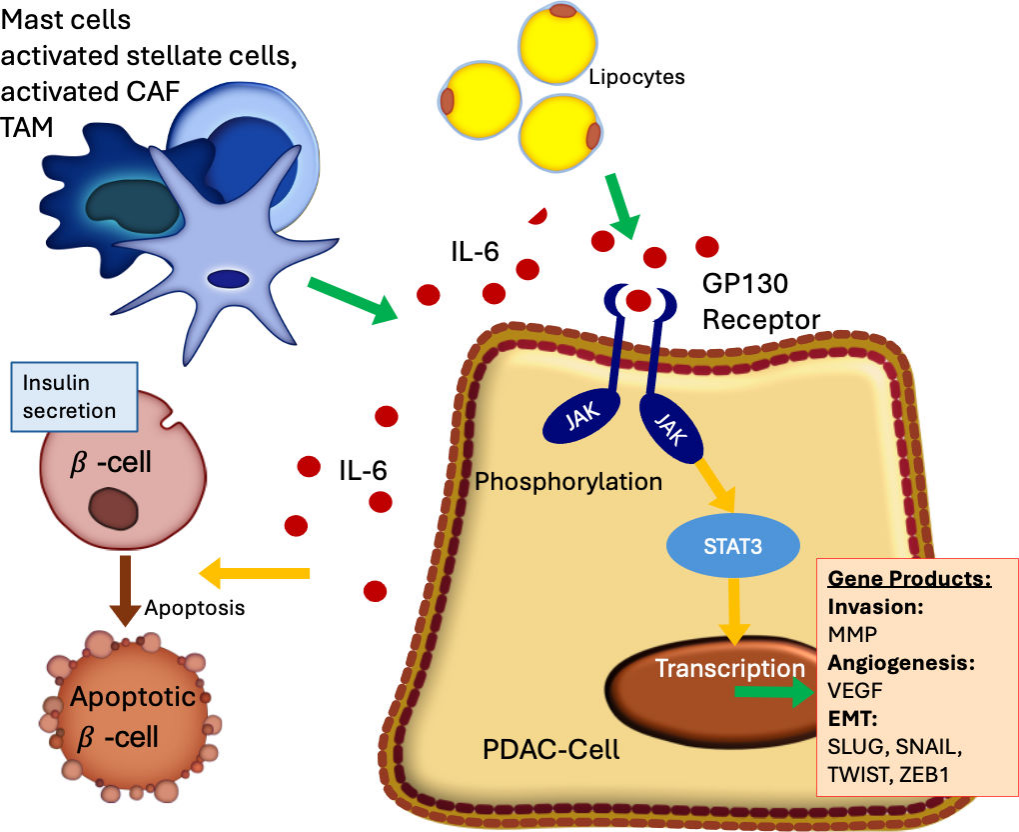
JAK-STAT3 pathways in pancreatic cancer. CAF: cancer-associated fibroblast; EMT: epithelial-mesenchymal transition; IL-6: interleukin 6; GP130: glycoprotein 130; JAK: Janus kinase; MMP: matrix metalloproteinase; PDAC: pancreatic ductal adenocarcinoma; STAT3: signal transducer and activator of transcription 3; TAM: tumor-activated macrophages; VEGF: vascular endothelial growth factor.

The proinflammatory cytokine IL-6 plays a pivotal role in tumorigenesis by activating the JAK-STAT3 signaling cascade. Upon binding to its IL-6 receptor α (IL-6Rα) on the cell surface, IL-6 induces dimerization with the coreceptor GP130, leading to phosphorylation of JAKs and subsequent activation of STAT3. Phosphorylated STAT3 translocates to the nucleus, where it promotes the transcription of genes involved in cell survival and proliferation. Recent findings have identified integrin β3 as a gene product associated with prognosis [9]. Moreover, IL-6/STAT3 signaling has been implicated in the progression from pancreatic intraepithelial neoplasia to PDAC [10]. IL-6 also contributes to the activation of cancer-associated fibroblasts via pancreatic stellate cells, key components of the tumor stroma [11]. Additionally, PDAC cells appear to exploit IL-6 signaling to prime the liver microenvironment for metastatic colonization [10]. These insights suggest that targeting the IL-6/GP130/JAK/STAT3 axis may offer a promising therapeutic strategy for pancreatic cancer. A phase 1 clinical trial has been initiated [12], evaluating a combination therapy comprising trametinib (mitogen-activated extracellular signal-regulated kinase [MEK] inhibitor), ruxolitinib (JAK2/STAT3 inhibitor), and retifanlimab (programmed cell death protein 1 [PD-1] inhibitor) [13-15]. Figure 1 shows the relevance of the pathway.

Pancreatic cancer is increasingly recognized for its impact on glycemic metabolism, with inflammatory cross talk proposed as a key underlying mechanism. Tumor-derived cytokines, notably IL-6 and tumor necrosis factor α (TNF-α), contribute to adipose tissue inflammation, impairing insulin sensitivity. TNF-α interferes with insulin receptor signaling in adipocytes, potentially promoting lipolysis and elevating levels of nonesterified fatty acids [16], which are commonly observed in obesity and type 2 diabetes, and are known to exacerbate insulin resistance and induce β-cell toxicity [17]. A study by Fogar et al [18] demonstrated a correlation between portal vein IL-6 levels and fasting serum glucose concentrations. Furthermore, preclinical evidence indicates that IL-6/GP130/JAK/STAT3 pathway activation upregulates autotaxin expression in adipocytes, while genetic suppression of autotaxin enhances insulin sensitivity and reduces hepatic steatosis in knockout mice compared to wild-type controls [19].

Inflammatory signaling within the tumor stroma is also implicated in chemotherapy resistance and disease progression. Specifically, the IL-6/GP130/JAK/STAT3 axis has been associated with cancer cachexia [20] and neural invasion [21]. Several therapeutic agents targeting this pathway have been proposed for pancreatic cancer treatment [22]. Given the marked heterogeneity of pancreatic tumors, precision therapies are urgently needed. However, the prevalence and clinical relevance of IL-6/GP130/JAK/STAT3 pathway activation in patients with pancreatic cancer remain poorly defined. Moreover, it is unclear whether pathway activation correlates with metabolic dysregulation or the presence of cachexia [23].

### Aim of This Study

This study aims to evaluate the activation of the IL-6/GP130/JAK/STAT3 signaling pathway in previously collected tissue samples—including pancreatic biopsies, surgical specimens, and liver biopsies—from patients diagnosed with pancreatic adenocarcinoma. The study will quantify pathway activation and assess its association with tumor stage, overall survival, and metabolic parameters, including the requirement for oral antidiabetic agents, insulin therapy, and hemoglobin A_1c _levels.

## Methods

### Recruitment

#### Overview

**Figure 2.**
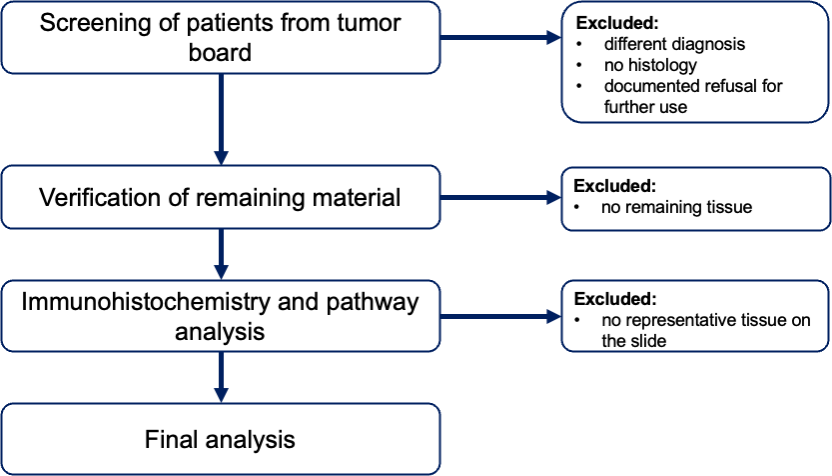
Inclusion process.

A retrospective cross-sectional study will be performed, including patients who were treated in our hospital between 2010 and 2024. We will use the existing clinical database of the Hospital Freiburg/Fribourg (HFR) to identify patients who received an operation for pancreatic adenocarcinoma and patients with advanced pancreatic cancers who could not undergo a resection and for which histological material is available. The data for patients with pancreatic adenocarcinoma will be obtained from the HFR database. The histological material (pancreas biopsies, surgical specimens, or liver biopsies) comes from the same patients and is currently stored in the HFR-affiliated laboratory Promed. Figure 2 shows the inclusion process.

#### Inclusion Criteria

All patients with a histological diagnosis of pancreatic cancer (PDAC) where sufficient histological material for immunohistochemistry analysis is available will be included, and the required clinical data will be recorded.

#### Exclusion Criteria

Patients will be excluded if there is documented refusal of further use of clinical samples or data.

### Materials

#### Samples

We will use previously taken histology samples (either after pancreatic resection, biopsies of liver metastasis, or endoscopic ultrasound–guided pancreas biopsies). The different origins of samples will be considered in the analysis. The samples were stored as tissue blocks in paraffin at Promed laboratory. They will be brought as slides to the core facility at École Polytechnique Fédérale de Lausanne (EPFL).

#### Immunohistochemistry

Immunohistochemistry will be performed using the Discovery Ventana ULTRA (Indianapolis, IN) system at the Histology Core Facility, EPFL, Lausanne, Switzerland (Phospho-STAT3 (Tyr705) (D3A7) XP rabbit monoclonal antibody [mAb]; Cell Signaling Technology, Leiden, The Netherlands). Stained slides will be scanned and images provided for activation analysis.

#### Examined Parameters

##### Primary End Point

The primary end point was IL-6/GP130/JAK/STAT3 pathway activation measured by immunohistochemistry (Phospho-STAT3 (Tyr705) (D3A7) XP rabbit mAb) in both tumor and stoma (where available) in percent. The activation will be measured using QuPath image software (version 0.5.1; Bankhead et al).

##### Secondary End Points

Clinical features—including tumor stage, surgical resection status, overall survival from the time of diagnostic biopsy, obesity, prior weight loss, presence of cachexia, and glucose metabolism disturbances—will be analyzed for their association with activation of the IL-6/GP130/JAK/STAT3 signaling pathway. Glucose metabolism will be assessed using a composite definition of prediabetes/diabetes based on hospital-recorded diagnoses, elevated hemoglobin A_1c_ or glucose values (including fasting glucose), and the use of antidiabetic medications. Additional comorbidities, such as rheumatic or autoimmune diseases, will also be considered in the analysis.

### Statistical Analysis

#### Power

We hypothesize that patients exhibiting strong activation of the IL-6/GP130/JAK/STAT3 pathway have a poorer prognosis compared to those with lower activation levels. Assuming a difference in pathway activation of 5% versus 10% (SD 10%) between patients who underwent surgical resection (early-stage disease) and those who did not (advanced-stage disease), a sample size of 84 patients per group would yield 90% statistical power, while 104 patients per group would achieve 95% power.

For our second primary outcome—the association between IL-6 pathway activation and diabetes—we will treat diabetes as a dichotomous variable and pathway activation as a continuous variable. Based on an expected difference in activation of 8% versus 11% (SD 7%), with higher activation in patients with diabetes, a sample size of 98 patients per group would provide 85% power. Given that approximately 50% of patients with pancreatic cancer are reported to have diabetes, this sample distribution appears feasible, though not guaranteed.

Sample size calculations were performed using MedCalc [24]. As complete clinical data may not be available for all patients, each analysis will be conducted using both complete-case and imputed datasets.

#### Evaluation Outcomes

Percentages of IL-6 pathway activation and measured clinical features will be reported. Means of values (pathway activation, laboratory values) will be compared. Survival differences between patients with strong versus weak pathway activation will be compared in a Cox hazard analysis. To determine the optimal cutoff, we will perform a threshold analysis. Hazard ratios (between patients with weak and with strong pathway activation) will be calculated for all examined metabolic parameters to investigate a possible association between pathway activation and diabetes. A regression model incorporating demographic, clinical, and pathway-related parameters will be fitted, with 95% CIs. The number of cases with missing data for all analyses will be reported.

### Ethical Considerations

This project was reviewed and approved by the Ethics Committee of Bern, Switzerland (project 2024‐01215).

#### Informed Consent, Further Use Without Consent

Since 2020, patients at HFR have provided general consent for the secondary use of their clinical data and biological material. However, patients diagnosed before this date did not provide such consent. Given the high lethality of pancreatic cancer, the vast majority of eligible patients are now deceased, making it impossible to obtain informed consent retrospectively. An exemption from informed consent was therefore requested and granted in accordance with Article 34 of the Swiss Human Research Act [25]. This exemption was justified because the anticipated gain in scientific knowledge offers significant potential benefit to patients and society, and that such findings could not be obtained without access to data from individuals who are deceased. In this context, the societal interest in advancing research was deemed to outweigh the individual interests of the affected patients.

#### Data Protection

##### Uncoded Data, Coding, and Storage of the Key

Clinical data will be transcribed from the hospital database and pseudonymized by authors CM and FS. Each patient will be assigned a unique code, and a separate key document linking codes to patient identities will be created and securely stored under double lock by the principal investigator. All analyses will be conducted using coded data in accordance with this study protocol and in full compliance with applicable data protection regulations.

##### Information on the Storage of Data and Samples

Clinical data will be extracted from both paper and electronic medical records and entered into a secure, password-protected trial database (REDCap). All personally identifiable information—including names, addresses, exact dates of birth, and hospital patient numbers—will be stored separately from the study dataset. Digital documents will be password protected, and physical records will be securely stored in a restricted-access research office.

Histological samples will be identified and deidentified by the laboratory Promed, replacing patient names with unique numerical codes. These samples will be prepared and sent to EPFL for immunohistochemical staining. Upon completion, the stained slides will be returned to our laboratory for assessment of pathway activation levels. Any remaining material will be returned to Promed for secure storage.

### Risk-Benefit Assessment

This project is expected to enhance our understanding of the role of IL-6/GP130/JAK/STAT3 pathway activation and its histological patterns in shaping the clinical phenotype of pancreatic adenocarcinoma, particularly its impact on glucose metabolism. These insights may contribute to the development of more targeted therapeutic strategies and help identify patient subgroups that could benefit from an IL-6 receptor blockade as part of their treatment regimen.

There is no risk to individual patients, as the study relies exclusively on previously collected routine clinical data and archived biopsy material. All data will be pseudonymized before analysis, and study results will not influence patient care.

The risk of inadvertent patient identification is minimal. Access to uncoded data is restricted to trained medical personnel—specifically, the three named investigators at HFR and the pathologist at Promed—who are bound by professional confidentiality through both their employment and the research framework. All digital files will be password protected, and physical documents will be stored securely in restricted-access facilities. This project is fully compliant with Swiss legal and ethical standards.

### Risk of Bias

To minimize the risk of biased conclusions due to nonrepresentative sampling, we will include all patients diagnosed with pancreatic cancer at HFR, applying the least restrictive exclusion criteria possible. As HFR is the only hospital in the canton of Fribourg with an oncology department, the vast majority of patients with pancreatic cancer in the region receive care at this institution. Consequently, only a small number of patients who seek treatment outside the canton are expected to be excluded, supporting the representativeness of our sample.

## Results

This project is supported by two research grants from the Hospital of Fribourg (HFR 2/2021 and 4/2022). As of October 12, 2025, clinical records from 150 patients have been included. Screening of additional patient records from 2024 to 2025 is ongoing.

## Discussion

### Expected Findings

We hypothesize that IL-6/GP130/JAK/STAT3 pathway activation is comparable between tumor tissue and adjacent stromal tissue. Furthermore, we expect that patients with stronger pathway activation will exhibit poorer prognosis, more significant weight loss, and greater or more recent disturbances in glucose metabolism. We do not anticipate an association between pathway activation and long-standing diabetes. Potential confounding factors include pancreatitis and other concurrent inflammatory conditions, such as pneumonia, which may independently influence pathway activation and metabolic parameters.

### Strengths and Weaknesses of This Study

A major strength of this study is that it represents only the second investigation of IL-6/GP130/JAK/STAT3 pathway activation in a substantial number of pancreatic cancer patient samples, following the recent work by Campos et al [9]. While the Campos study focused primarily on mechanistic insights, our study emphasizes the correlation between pathway activation and clinical symptoms and outcomes, offering novel perspectives on its relevance in patient care. We will also assess the presence of pancreatitis and antibiotic use, which may help exclude infectious diseases as sources of inflammation.

A potential limitation is the sample size, which may be insufficient to detect statistically robust associations across all variables. Additionally, the study design does not allow for longitudinal analysis, as only a single tissue sample per patient is available. Therefore, we cannot determine whether pathway activation varies over the course of disease progression.

### Conclusion

This study contributes important new insights into the IL-6/GP130/JAK/STAT3 pathway activation in patients with PDAC. Preliminary findings suggest that patients with advanced-stage disease exhibit higher levels of pathway activation in both tumor and adjacent stromal tissue. These results support the hypothesis that stromal activation mirrors tumor activation and may play a role in disease progression. To further disentangle the influence of patient or tumor genetics from disease dynamics, longitudinal sampling would be necessary to assess changes in pathway activation over time.

## Abbreviations

EPFL: École Polytechnique Fédérale de Lausanne
ESPAC: European Study Group for Pancreatic Cancer
GP130: glycoprotein 130
HFR: Hospital Freiburg/Fribourg
IL-6: interleukin 6
IL-6Rα: interleukin 6 receptor α
JAK: Janus kinase
mAb: monoclonal antibody
MEK: mitogen-activated extracellular signal-regulated kinase
PD-1: programmed cell death protein 1
PDAC: pancreatic ductal adenocarcinoma
STAT3: signal transducer and activator of transcription 3
TNF-α: tumor necrosis factor α
